# A Standard of Knowledge for the Professional Practice of Toxicology

**DOI:** 10.1289/ehp.1408643

**Published:** 2015-03-17

**Authors:** Janis E. Hulla, Lewis B. Kinter, Bruce Kelman

**Affiliations:** 1 Environmental Engineering Branch, Sacramento District, U.S. Army Corps of Engineers, Sacramento, California, USA; 2 AstraZeneca Pharmaceuticals, Drug Safety & Metabolism, Wilmington, Delaware, USA; 3 Veritox Inc., Redmond, Washington, USA

## Abstract

**Background:**

Employers, courts, and the general public judge the credibility of professionals based on credentials such as academic degrees, publications, memberships in professional organizations, board certifications, and professional registrations. However, the relevance and merit of these credentials can be difficult to determine objectively. Board certification can be a reliable indicator of proficiency if the certifying organization demonstrates, through regularly scheduled independent review, that its processes meet established standards and when a certificate holder is required to periodically demonstrate command of a body of knowledge that is essential to current professional practice.

**Objective:**

We report herein a current Standard of Knowledge in general toxicology compiled from the experience and opinions of 889 certified practicing professional toxicologists.

**Discussion:**

An examination is the most commonly used instrument for testing a certification candidate’s command of the body of knowledge. However, an examination-based certification is only creditable when the body of knowledge, to which a certification examination tests, is representative of the current knowledge, skills, and capabilities needed to effectively practice at the professional level. Thus, that body of knowledge must be the current “Standard of Knowledge” for the profession, compiled in a transparent fashion from current practitioners of the profession.

**Conclusion:**

This work was conducted toward ensuring the scientific integrity of the products produced by professional toxicologists.

**Citation:**

Hulla JE, Kinter LB, Kelman B. 2015. A Standard of Knowledge for the professional practice of toxicology. Environ Health Perspect 123:743–748; http://dx.doi.org/10.1289/ehp.1408643

## Introduction

*Background.* Academic training programs in the field of general toxicology have undergone dramatic changes in past decades and the content of core instruction and research experiences varies widely across global regions, academic programs, and institutions (Brock et al. 2009). Approximately 50% of the International Union of Toxicology’s (IUTOX) 51 member societies are involved in some type of credentialing effort at the national level; however, definitions vary greatly from one group to another. Some professional societies provide registration for their members; others provide certification based upon successful completion of an examination and/or peer review of professional credentials. There is a demand for a credentialing process that is recognized and accepted worldwide. For toxicologists, the courts, and the public in general, an internationally recognized certification is of value to the extent those stakeholders can be confident that the credential demonstrates currency and command of both breadth and appropriate depth of relevant knowledge and current professional experience.

The benefits of certification to an individual professional toxicologist include advantages in the job market and career development. This is made clear by the results of the published series of eight triennial salary surveys that were circulated to members of the Society of Toxicology, the American College of Toxicology, and 23 other professional organizations. These surveys have consistently shown that certified toxicologists have higher levels of compensation (Gad and Sullivan 2013).

The largest organization certifying proficiency in general toxicology based in part upon a written examination is the American Board of Toxicology (ABT). The ABT was incorporated in the District of Columbia (USA) on 17 April 1979 as a self-sustaining not-for-profit corporation (ABT 2011). The mission of the ABT is to identify, maintain, and evolve a standard for professional competency in the field of toxicology. The processes through which the ABT has established a globally recognized credential in toxicology includes a qualifying review of candidates’ professional credentials and a three-part certification examination. The examination is the tool by which the ABT tests prequalified candidates’ competency with respect to a current Standard of Knowledge of toxicology. In this context, “Standard of Knowledge” refers to the knowledge and skills needed to practice toxicology at the professional level, across a broad swath of professional toxicological roles; it is a “living standard” and must be periodically updated to reflect the evolution of the practice of toxicology and current knowledge status. The ABT’s processes include periodic independent review of its certification program. Historically, the independent reviews have focused on ABT’s compliance with applicable standards established by the National Commission for Certifying Agencies (NCCA 2004). Based on the findings of these reviews, changes have been implemented to better ensure the quality of the examination, maintenance of its integrity, and relevance to current professional practice of toxicology.

*Objective.* The ABT Board of Directors recently elected to update its professional Standard of Knowledge. In developing the updated Standard, the Board members empowered a committee of currently practicing toxicologists to design and implement a process to return information useful in defining the skills and knowledge taught in academic institutions and acquired through the professional practices of toxicology. To our knowledge, this is the first such effort to define knowledge domains and specific elements based on input from professional toxicologists practicing across a broad spectrum of employment sectors, as assessed from the perspective of current practicing professional toxicologists.

## Method

Toward meeting a standard established by the NCCA, a committee of ABT diplomates developed a survey of specific knowledge domains and, within each domain, specific knowledge elements essential to effective employment/practice as a professional toxicologist. The survey participants were asked, from the perspective of their personal professional practice/experience, to identify and rank the level of importance of each knowledge element in order of importance as:

Working knowledge, considered most important
General knowledge, less important
Unable to determine necessity, still less important
Not needed, considered unimportant in the general practice of toxicology.

For the purpose of the survey, working knowledge was defined as a demonstrated ability to apply concepts; select and apply specific testing strategies; calculate, transform, and analyze data; interpret case studies; and associate specific mechanisms or patterns of toxicity with specific elements (e.g., metals), chemicals, and groups of chemicals (e.g., mixtures). General knowledge was defined as knowledge of the relevant definitions, specific mechanisms of action, and contexts, including an ability to apply the general concepts and recognize major data-response patterns and an ability to interpret data and make conclusions.

The survey instrument was initially drafted from an amalgamation of tables of contents of several current textbooks in toxicology, and subsequently refined by discussions among committee members to create a pilot survey. Circulation of the pilot survey was limited to current and past members of the ABT Board of Directors and was administered in the first quarter of 2013. In the second quarter of 2013, a finalized survey was more broadly circulated to the entire ABT diplomate community. In both the pilot and finalized surveys, participants were asked to add and rank any additional knowledge domains and elements missing from the survey they deemed relevant to their professional toxicology role. The results of the combined pilot and revised surveys were used to extract the current Standard of Knowledge for proficiency in general toxicology across all participating toxicological roles, based on at least 50% required competence levels of either working or general knowledge by the surveyed diplomates. The Army Human Research Protections Office determined that the surveys performed for this report did not constitute human subjects research.

## Results

**Figure 1:**
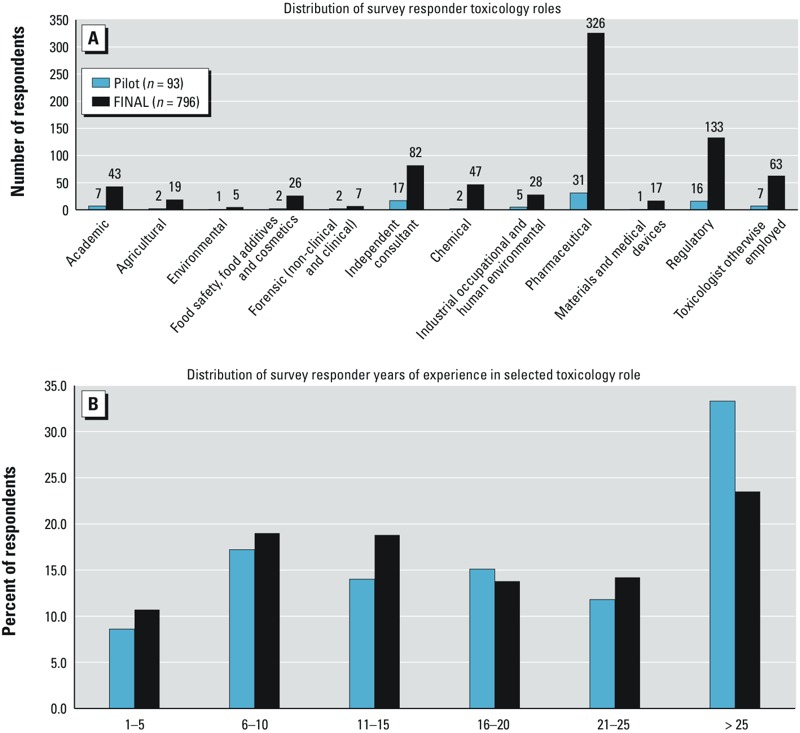
(*A*) Distribution of the survey participants’ employment sectors. (*B*) Distribution of participants’ years of employment as a toxicologist.

Thirty-four percent (889 of 2,606) of the Diplomates of the ABT completed the survey. This included the 93 who participated in the pilot survey and 796 from the ABT-wide survey. By employment sector, respondents were 40.1% Pharmaceutical, 16.8% Regulatory, 11.1% Consultant, 7.9% “other,” 5.9% Industrial, 5.6% Academic, 5.5% Chemical, 3.7% Occupational, 3.1% Food Safety, 2.4% Agricultural, 2.0% Medical Devices, 1.0% Forensics, and 0.7% Environmental (Figure 1A). Participants with > 25 years of experience were the largest percentage of respondents (Figure 1B).

**Figure 2:**
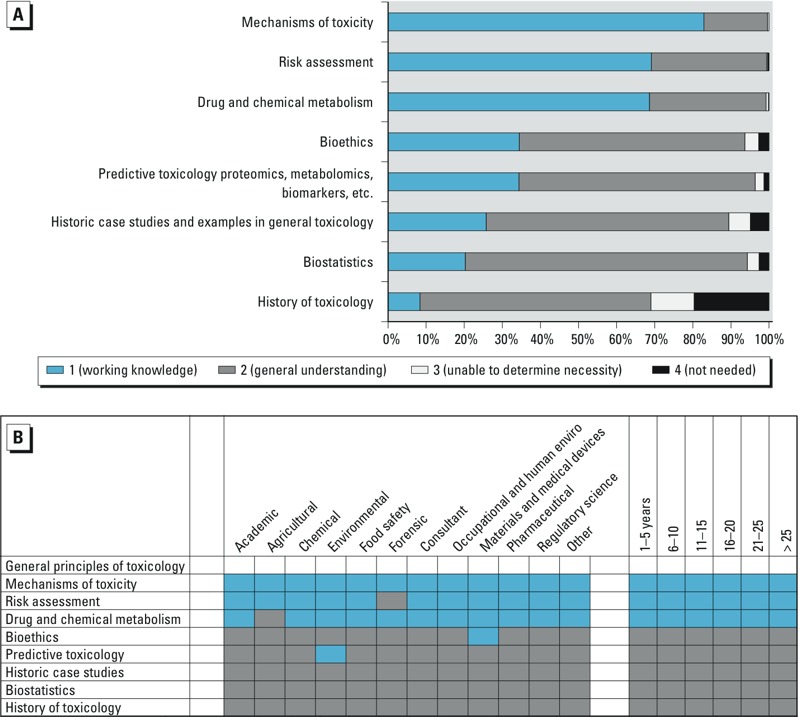
(*A*) Ranking of importance of information related to the knowledge domain “General principles of toxicology.” (*B*) Each knowledge element within the domain ranked by the demographics of specific employment sectors (left) and years of experience of the survey participants (right).

**Figure 3:**
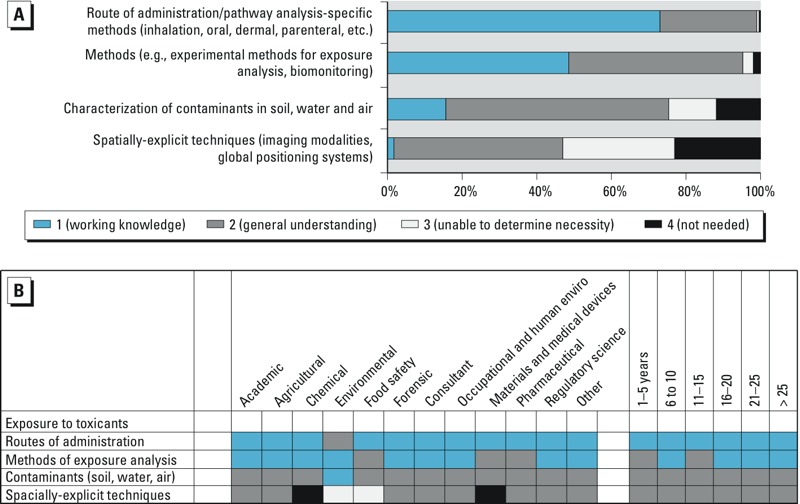
(*A*) Ranking of importance of information related to the knowledge domain “Exposure to toxicants.” (*B*) Each knowledge element within the domain ranked by the demographics of specific employment sectors (left) and years of experience of the survey participants (right).

**Figure 4:**
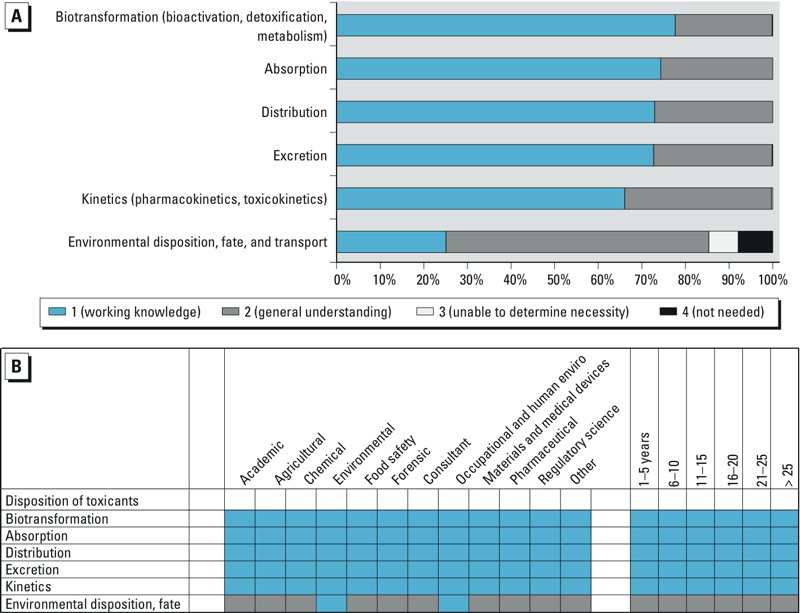
(*A*) Ranking of importance of information related to the knowledge domain “Disposition of toxicants.” (*B*) Each knowledge element within the domain ranked by the demographics of specific employment sectors (left) and years of experience of the survey participants (right).

**Figure 5:**
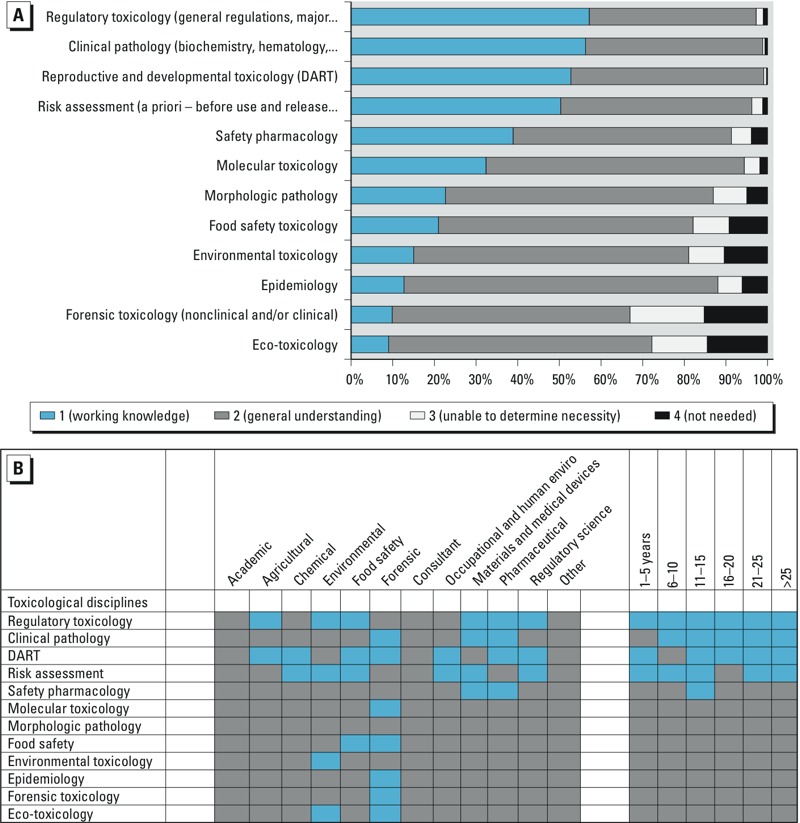
(*A*) Ranking of importance of information related to the knowledge domain “Toxicological disciplines.” (*B*) Each knowledge element within the domain ranked by the demographics of specific employment sectors (left) and years of experience of the survey participants (right). DART, developmental and reproductive toxicology.

The survey identified nine domains of toxicological knowledge: *1*) General principles of toxicology, *2*) Exposure to toxicants, *3*) Disposition of toxicants, *4*) Toxicological disciplines, *5*) Target organ systems toxicology, *6*) Non-organ directed toxicology, *7*) Toxic agents, *8*) Applications of toxicology, and *9*) Methods in toxicology. Each domain was divided into 4–14 knowledge elements (Table 1). How the survey participants ranked the individual knowledge elements is shown in Figures 2–5; (see also Supplemental Material, Figures S1–S5). In panel A of each of the figures, the length of the bar represents the proportion of the total responses at each level of importance: working knowledge, general understanding, not needed, or unable to determine. The total number of survey respondents is shown within the bars, where possible. Panel B of each figure shows how each knowledge element was ranked by the demographics of specific employment sectors and years of experience of the survey participants. Shown are the most frequent responses, generally > 50%, for each demographic group.

**Table 1:** Domains of toxicological knowledge and knowledge elements that define the Standard of Knowledge.

Working knowledge	General knowledge
General principles of toxicology
Drug and chemical metabolism	Bioethics (animal and human research)
Mechanisms of toxicity	Biostatistics
Risk assessment	Historic case studies
	History of toxicology
	Predictive toxicology
Exposure to toxicants
Methods (exposure analysis, biomonitoring)	Characterization of contaminants in soil, water, and air
Routes of administration/pathway-specific methods	Spatially specific techniques (imaging modalities, GPS)
Disposition of toxicants
Absorption	Environmental disposition, fate, and transport
Biotransformation
Distribution
Excretion
Kinetics
Toxicological disciplines
Clinical pathology	Ecological
Regulatory	Environmental
DART	Epidemiology
Risk assessment	Food safety
	Forensic
	Molecular toxicology
	Morphologic pathology
	Safety pharmacology
Target organ systems toxicology
Kidney/lower urinary tract	Blood
Liver	Bone and bone marrow
Lung/respiratory system	Endocrine
	Gastrointestinal
	Cardiac and vascular
	Immune
	Muscular
	Nervous
	Ocular, olfactory, and visual
	Skin and integument
	Reproductive organs
Non-organ directed toxicology
Chemical carcinogenicity	Direct-acting agents
Genetic and epigenetic	Nanotoxicology
	Phototoxicology
Toxic Agents
	Animal venoms and toxins
	Aromatic hydrocarbons
	Endocrine systems–disrupting compounds
	Gases, solvents, vapors, and particles
	Halogenated organic compounds
	Metals
	Mycotoxins
	Nanomaterials
	Pesticides
	Pharmaceuticals
	Phytotoxins
	Polyaromatic hydrocarbons
	Prokaryote toxins
	Radiation and radioactive materials
Applications of toxicology
Risk assessment: human, clinical	Analytical chemistry
Safety assessment: nonclinical	Clinical
	Devices
	Environmental risk assessment
	Food safety and food products
	Forensics
	Occupational
	Pharmaceuticals and biotechnology products
	Veterinary products
Methods in toxicology
*In vivo* methods	Adverse outcome pathway analyses
Study design	Clinical studies and population monitoring
	Environmental methods
	*In silico* methods
	*In vitro* methods
	Transcriptomics technologies
Abbreviations: DART, developmental and reproductive toxicology; GPS, global positioning system.	

Survey participants were asked to rank the importance of specific knowledge elements in the domain “General principles of toxicology.” The knowledge elements with the highest working knowledge needs were mechanisms of toxicity and risk assessment. History of toxicology was rated the lowest (Figure 2A). The evaluations of the results in terms of the respondents’ demographics are shown in Figure 2B. The data indicate that years of experience was not a factor in participants’ response that professional practice requires working knowledge of mechanisms of toxicity, risk assessment, and drug and chemical metabolism.

Survey participants were asked to rank the importance of knowledge elements in the domain “Exposure to toxicants.” The highest ranked area for working knowledge was route of administration. The lowest was spatially explicit techniques (Figure 3A). Participants at all levels of experience, with the exception of those working in the Environmental sector, responded that working knowledge of routes of administration was required in their professional practice (Figure 3B).

Survey participants were asked to rank the importance of knowledge elements within the domain “Disposition of toxicants.” The area highest ranked for working knowledge was biotransformation, and the lowest was environmental fate and transport (Figure 4A). Participants in each of the employment sectors and experience levels responded that working knowledge of biotransformation, absorption, distribution, excretion, and kinetics is required in their professional practice (Figure 4B).

Survey participants were asked to rank of importance of knowledge elements within the domain “Toxicological disciplines.” The discipline with the highest working knowledge ranking was regulatory toxicology. The lowest was ecotoxicology (Figure 5A). Participants at all levels of experience responded that working knowledge of regulatory toxicology is required in their professional practice. However, this response was not consistent across the employment sectors (Figure 5B).

Survey participants were asked to rank the importance of knowledge elements in the domain “Target organ systems toxicology.” Working knowledge of liver toxicity was the highest ranked, and musculature systems toxicity was lowest (see Supplemental Material, Figure S1A). Participants at each level of experience responded that working knowledge of liver toxicity is required in their professional practice (see Supplemental Material, Figure S1B).

Survey participants were asked to rank the importance of knowledge elements in the domain of “Non-organ directed toxicity”; working knowledge of chemical carcinogenesis ranked highest, and nanotoxicology ranked lowest (see Supplemental Material, Figure S2A). Participants at all levels of experience and in each of the employment sectors responded that working knowledge of chemical carcinogenesis is required in their professional practice (see Supplemental Material, Figure S2B).

Survey participants were asked to rank the importance of knowledge elements in the domain of “Toxic agents”; endocrine systems disruptors ranked highest for combined working and general knowledge, and prokaryote toxins were lowest (see Supplemental Material, Figure S3A). The demographic groups’ responses varied widely with respect to the working and general knowledge rankings (see Supplemental Material, Figure S3B).

Survey participants were asked to rank the importance of specific knowledge elements within the domain of “Applied toxicology”; safety assessment ranked highest for working knowledge, and forensics was lowest (see Supplemental Material, Figure S4A). Participants at each experience level responded that working knowledge of both safety assessment and risk assessment is required in their professional practice (see Supplemental Material, Figure S4B.

Survey participants were asked to rank the importance of knowledge elements in the domain “Methods”; study design ranked highest for working knowledge, and environmental methods ranked lowest (see Supplemental Material, Figure S5A). Each of the demographic groups, with the exception of the Environmental sector, responded that working knowledge of both study design and *in vivo* methods is required in the professional practice (see Supplemental Material, Figure S5B).

Eighty-five percent of the respondents indicated that there were no missing topics. The remaining responses included topics such as susceptible species and human subpopulations; genetics, genomics, and toxicogenomics; scientific and risk communication; risk management; employee management; and pharmacology, biopharmaceuticals, botanicals, and drug safety. These were evaluated and deemed either included in existing knowledge elements or not relevant to the Standard of Knowledge for general toxicology.

## Discussion

A global demand for standardization of processes for credentialing professional toxicologists is evidenced by the results of the IUTOX 2009 and 2011 surveys that showed high interest, among the IUTOX 51 affiliated societies and > 20,000 member toxicologists, in identifying a process through which toxicologists might achieve professional recognition (IUTOX 2014b). The IUTOX Toxicologist Recognition Task Force endorsed the concept that fundamental to achieving credibility for any professional credential is the transparency of the certifying organization’s source of funding, affiliations, criteria used to award the credential, the requirements that maintain currency of the credential, and independent review/audit processes. In this context, the Task Force has published information on the certifications and registrations bestowed by IUTOX membership organizations (IUTOX 2014a).

Further evidence of the societal demand for credible credentialed professionals is provided in the National Research Council’s (NRC) Committee on Science, Technology, Law, Policy and Global Affairs’ third edition of *Reference Manual on Scientific Evidence* (NRC 2011). The credentials recognized as “Expert Qualifications” for toxicologists are discussed in the chapter “Reference Guide to Toxicology” (Goldstein and Henifen 2011). Board certification by the ABT is specifically cited, as is membership in the Academy of Toxicological Sciences, Society of Toxicology, American College of Medical Toxicology, American Academy of Clinical Toxicologists, and several others. In Europe, many toxicologists have been admitted to national registries under the auspices of EUROTOX. It is also noteworthy that the National Toxicology Program’s (NTP) specifications for the conduct of toxicity studies state that it is desirable for their study directors to be certified by the ABT (NTP 2011).

A description of professional roles for toxicologists with required educational background, training, and experience that is used for certifying European Registered Toxicologists has been previously published by EUROTOX (Fowler and Galli 2007). Building on the efforts of EUROTOX, this Standard of Knowledge is based on survey data from professional toxicologists practicing across a broad spectrum of employment sectors (NCCA 2004).

The NCCA provides standards for assessment-based certificate programs (NCCA 2004). The NCCA’s Standard 10 states that certification programs

must analyze, define, and publish performance domains and tasks related to the purpose of the credential, and the knowledge and/or skill associated with the performance domains and tasks, and use them to develop specifications for the assessment instruments.

The survey of Diplomates of the ABT returned information on the relative importance of job-related knowledge elements within conventional knowledge domains applied across 12 different professional practices of toxicology. From these data, the committee derived a Standard of Knowledge. It is based on at least 50% required competence levels of either working or general knowledge by surveyed diplomates (Table 1).

This Standard of Knowledge is based on current input from practicing professionals who represent the diversity of toxicological job roles and has many applications. First and foremost, it provides an objective and relevant basis for evaluations of candidates seeking credentialing in general toxicology. The Standard provides an objective basis for potential candidates to assess their professional training, knowledge, and experience in general toxicology, and identify their strengths and development opportunities. The Standard also provides employers and other interested parties the bases to understand the meaning and significance of credentialing linked to that standard. The Standard is also a “living” standard: It is anticipated to change and require periodic updating to reflect new science and regulation, and evolution of the toxicological disciplines and professional job roles.

The Standard of Knowledge for general toxicology presented herein could be criticized in that the authors limited their survey to ABT diplomates. The rationale for this decision was to ensure that survey participants were current practitioners of toxicology at a professional level, a Diplomate of the American Board of Toxicology (DABT) certification criterion. As shown in Figure 1, the surveyed diplomates represented 12 different professional toxicology practices, most of which were represented by > 15 respondents. However, in spite of the large total number of respondents, certain professional practices of toxicology with < 15 respondents (e.g., environmental and forensic toxicology) may be underrepresented. Future iterations of this survey should consider how to ensure that all areas of professional practice are appropriately represented.

## Conclusions

The ABT Board of Directors applies information gleaned from surveys such as the one described herein, contents of textbooks used in leading academic toxicology programs, and sources of information on emerging toxicological science and technology to define a robust and current Standard of Knowledge. Each year, the ABT Board of Directors develops a new certification exam as an instrument to test the certification candidates’ command of the Standard of Knowledge. Further, to assure the integrity of the DABT certification, the ABT implements periodic independent review and audit of the Board’s technical and financial processes. The independent reviewers and auditors evaluate the ABT procedures for meeting well-recognized standards of operation specifically designed for organizations having the mission of professional credentialing. It is a fundamental goal of the ABT to raise the standards of professional practice globally, and the ABT Board of Directors encourages other organizations involved in credentialing professional toxicologists to adopt the processes and the Standard of Knowledge described herein. To our knowledge, this is the first Standard of Knowledge published for the profession of toxicology based on data from a broad range of practicing toxicologists.

## Supplemental Material

(404 KB) PDF
