# Supplementary material for: A Standard of Knowledge for the Professional Practice of Toxicology
Source: Environ Health Perspect. 2015 Mar 17;123(8):743–8. doi: 10.1289/ehp.1408643 (PMC4529009; doi:10.1289/ehp.1408643)
Supplement: (404 KB) PDF [file ehp.1408643.s001.acco.pdf]

Environ Health Perspect
doi: 10.1289/ehp.1408643

Note to Readers: ***EHP*** **strives to ensure that all journal content is accessible to all readers. However, some figures and Supplemental Material published in *EHP* articles may not conform to 508 standards due to the complexity of the information being presented. If you need assistance accessing journal content, please contact ehp508@niehs.nih.gov. Our staff will work with you to assess and meet your accessibility needs within 3 working days.**

Supplemental Material

A Standard of Knowledge for the Professional Practice of Toxicology

Janis E. Hulla, Lewis B. Kinter, and Bruce Kelman

Table of Contents

Figure S1. Target Organ Toxicology. A: Distribution of rankings for the knowledge domain, Target Organ Toxicology. B: Majority (≥50%) ranking of knowledge elements within the domain by the specific employment sectors (left) and years of experience of the survey participants (right).

Figure S2. Non-Organ Directed Toxicity. A: Distribution of rankings for the knowledge domain, Non-Organ Directed Toxicity. B: Majority (≥50%) ranking of knowledge elements within the domain by the specific employment sectors (left) and years of experience of the survey participants (right).

Figure S3. Toxic Agents. A: Distribution of rankings for the knowledge domain, Toxic Agents. B: Majority (≥50%) ranking of knowledge elements within the domain by the specific employment sectors (left) and years of experience of the survey participants (right).

Figure S4. Applications of Toxicology. A: Distribution of rankings for the knowledge domain, Applications of Toxicology. B: Majority (≥50%) ranking of knowledge elements within the domain by the specific employment sectors (left) and years of experience of the survey participants (right).

Figure S5. Methods in Toxicology. A: Distribution of rankings for the knowledge domain, Methods in Toxicology. B: Majority (≥50%) ranking of knowledge elements within the domain by the specific employment sectors (left) and years of experience of the survey participants (right).

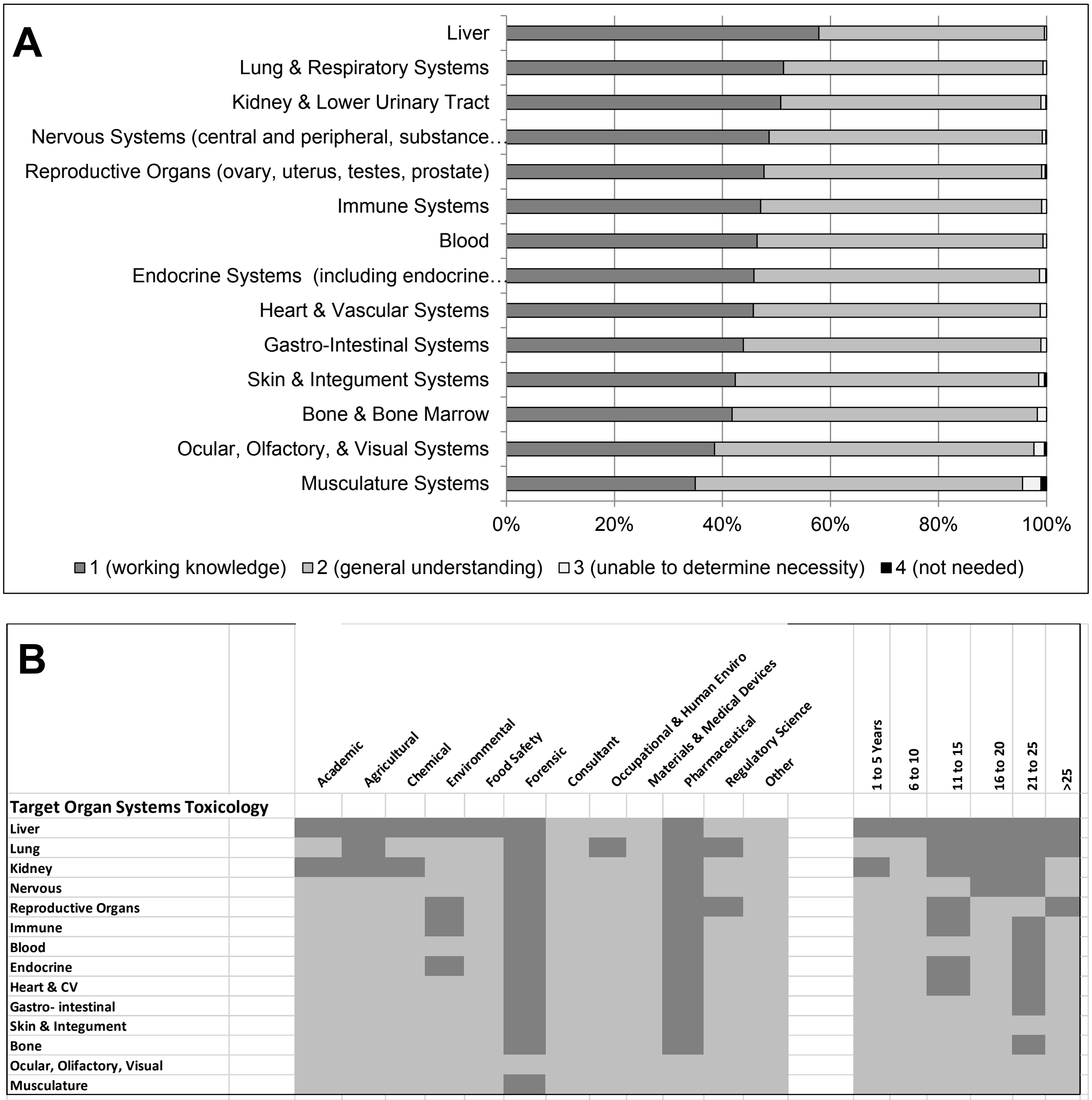

Figure S1. Target Organ Toxicology. A: Distribution of rankings for the knowledge domain, Target Organ Toxicology. B: Majority (≥50%) ranking of knowledge elements within the domain by the specific employment sectors (left) and years of experience of the survey participants (right).

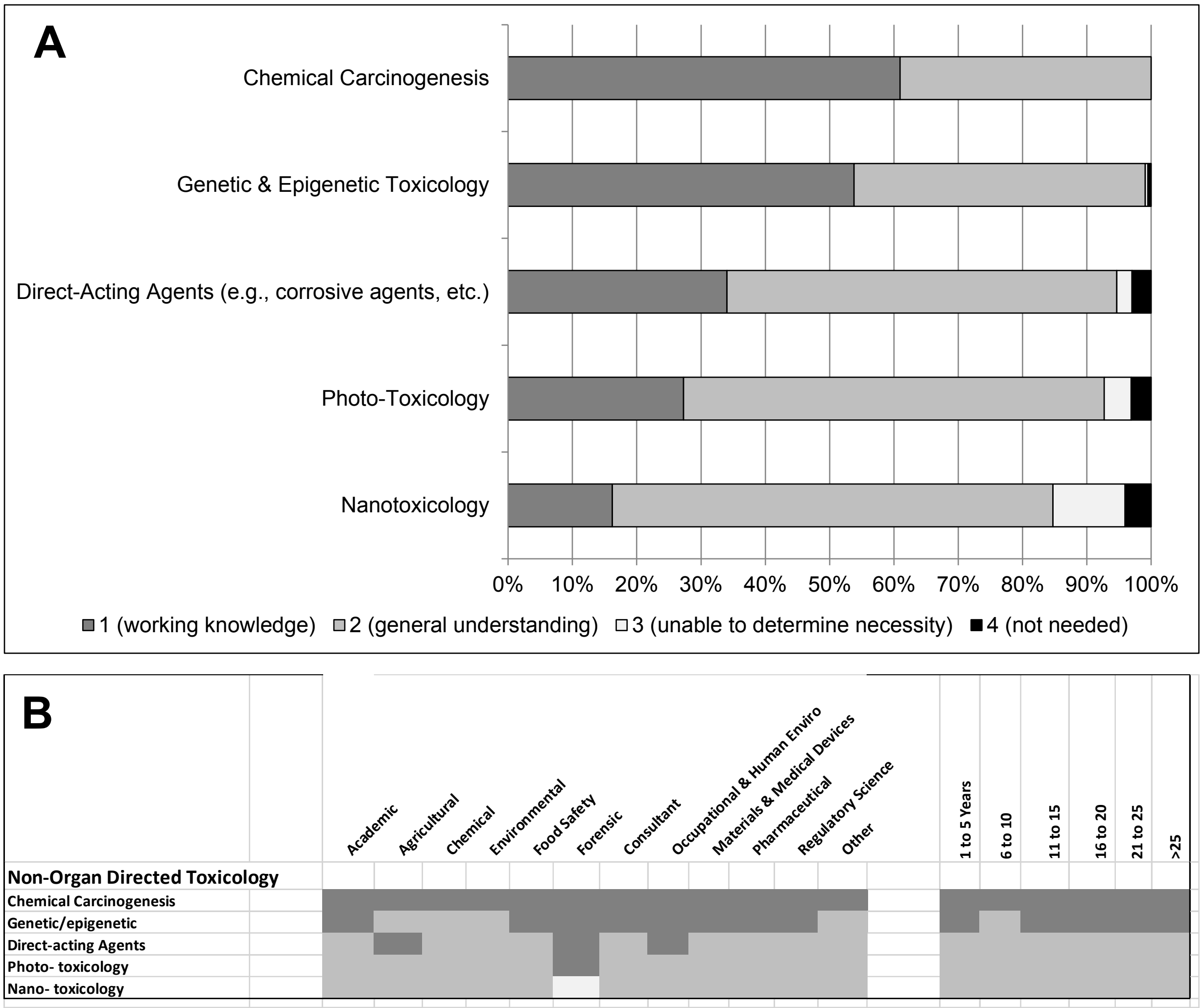

Figure S2. Non-Organ Directed Toxicity. A: Distribution of rankings for the knowledge domain, Non-Organ Directed Toxicity. B: Majority (≥50%) ranking of knowledge elements within the domain by the specific employment sectors (left) and years of experience of the survey participants (right).

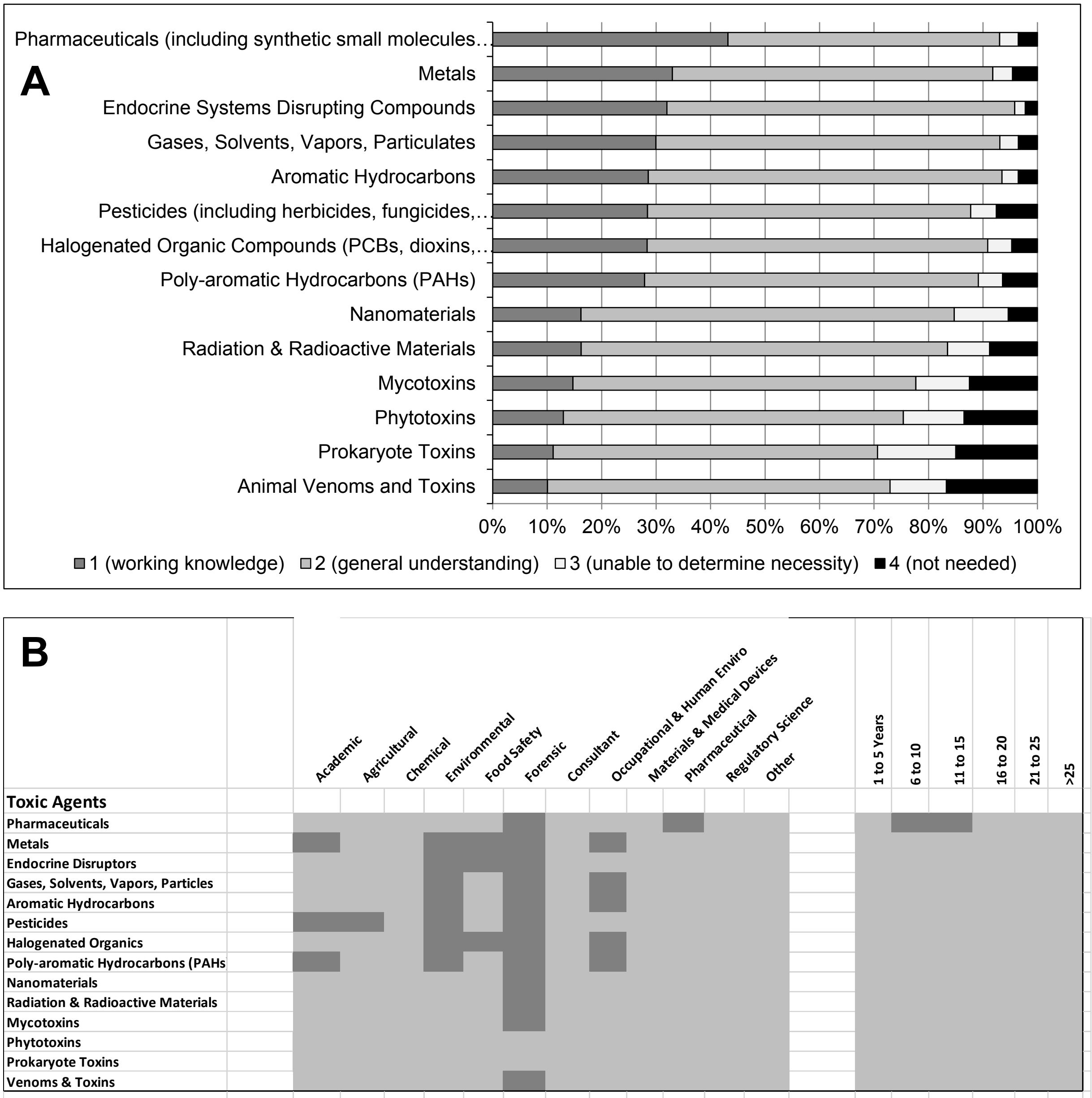

Figure S3. Toxic Agents. A: Distribution of rankings for the knowledge domain, Toxic Agents. B: Majority (≥50%) ranking of knowledge elements within the domain by the specific employment sectors (left) and years of experience of the survey participants (right).

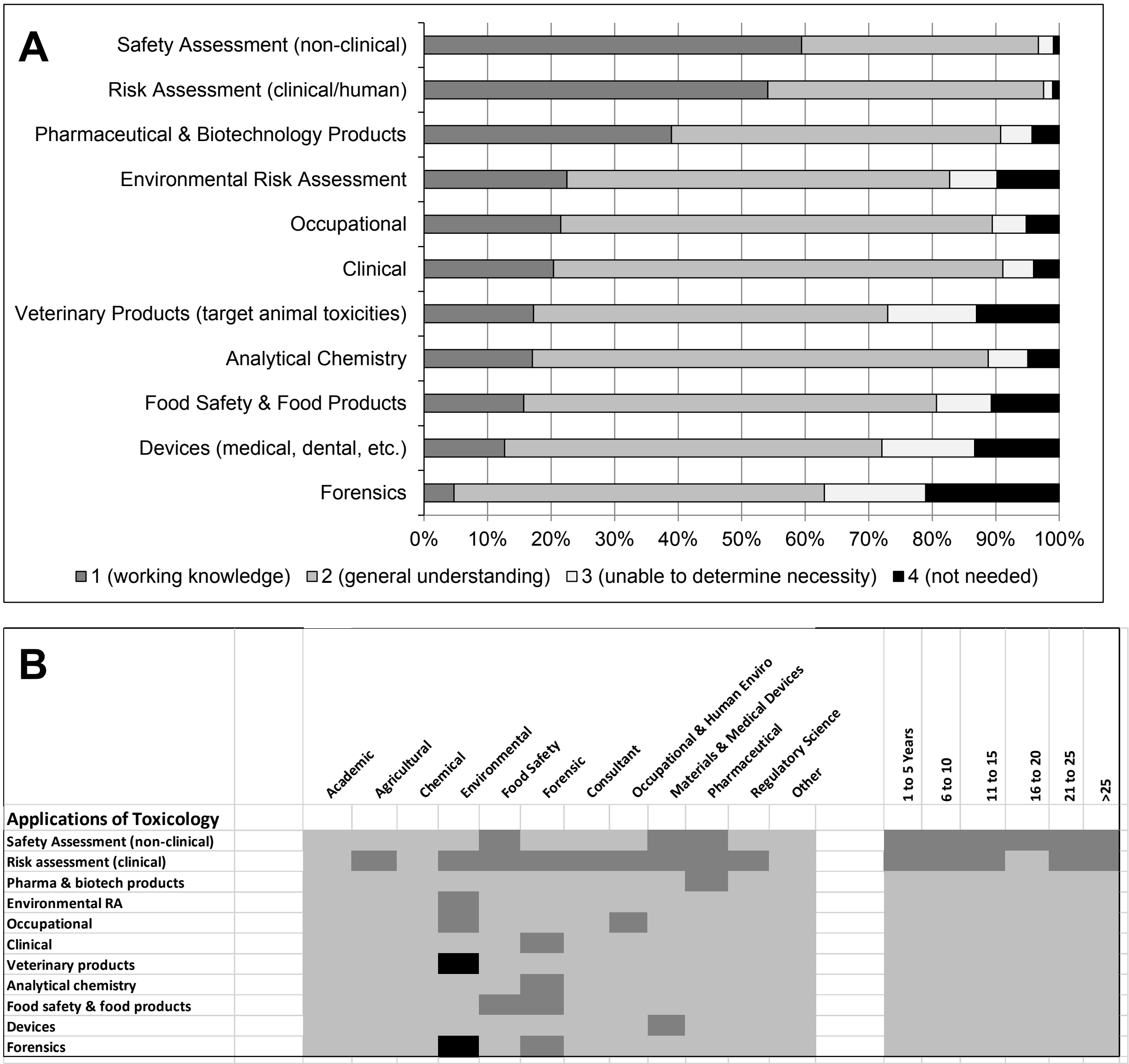

Figure S4. Applications of Toxicology. A: Distribution of rankings for the knowledge domain, Applications of Toxicology. B: Majority (≥50%) ranking of knowledge elements within the domain by the specific employment sectors (left) and years of experience of the survey participants (right).

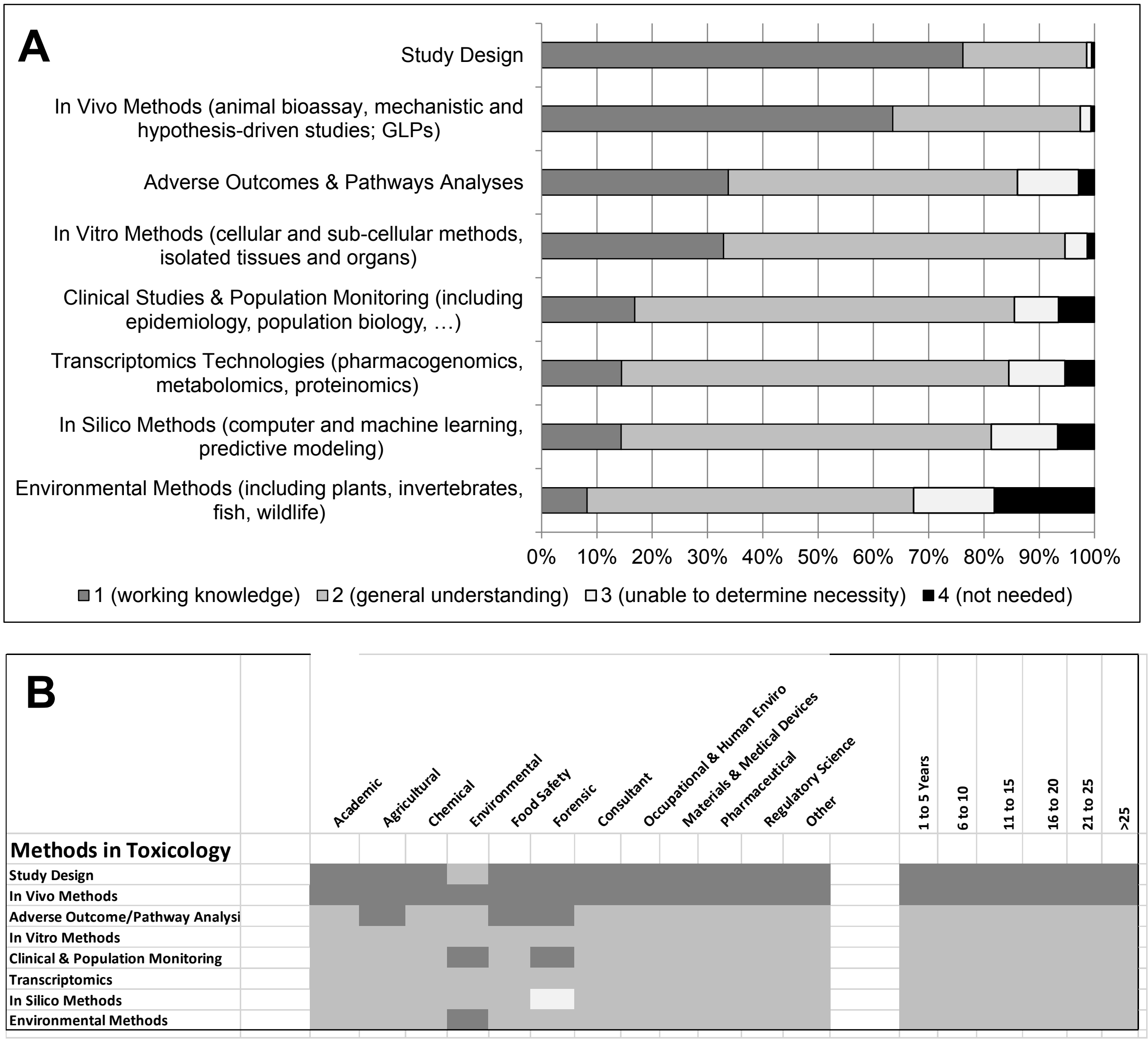

Figure S5. Methods in Toxicology. A: Distribution of rankings for the knowledge domain, Methods in Toxicology. B: Majority (≥50%) ranking of knowledge elements within the domain by the specific employment sectors (left) and years of experience of the survey participants (right).
